# Comparing the effects of isoflurane and pentobarbital on the responses of cutaneous mechanoreceptive afferents

**DOI:** 10.1186/1471-2253-13-10

**Published:** 2013-05-10

**Authors:** Ju-Wen Cheng, Alison I Weber, Sliman J Bensmaia

**Affiliations:** 1 Department of Physical Medicine and Rehabilitation, Chang Gung Memorial Hospital at Linkou, Taoyuan, Taiwan; Department of Organismal Biology and Anatomy, University of Chicago, Chicago, IL, USA; 2 Department of Organismal Biology and Anatomy, University of Chicago, Chicago, IL, USA

**Keywords:** Mechanoreceptive afferents, Anesthesia, Isoflurane, Pentobarbital, Vibration, Entrainment

## Abstract

**Background:**

While pentobarbital has been used extensively in neurophysiological experiments investigating activity in peripheral nerves, it has fallen out of favor as an anesthetic because of safety concerns and is often replaced with isoflurane. However, the effects of isoflurane on the excitability of mechanoreceptive afferents have yet to be conclusively elucidated.

**Methods:**

To fill this gap, we collected extracellular single-unit recordings of cutaneous mechanoreceptive afferents from the sciatic nerve of 21 rats during vibratory stimulation of the hindpaw. We then compared the strength and temporal structure of the afferent response measured under pentobarbital and isoflurane anesthesia.

**Results:**

We found that the strength and temporal structure of afferent responses were statistically equivalent whether these were evoked under isoflurane or pentobarbital.

**Conclusions:**

We conclude that, if these two anesthetics have any effect on the responses of mechanoreceptive afferents, their effects are indistinguishable.

## Background

Pentobarbital was the most commonly used anesthetic agent in neuroscience experiments in 2000–2001, but its use decreased from 33% in 2000–2001 to 16% in 2005–2006, while that of isoflurane and ketamine/xylazine increased (2 to 16% and 15 to 31%, for isoflurane and ketamine/xylazine respectively) [1]. Previous studies have shown that these anesthetics cause disparate changes in cortical responses, but their effects on the sensory periphery, and on mechanoreceptive afferents in particular, remain to be conclusively elucidated. To fill this gap, we wished to compare the effects of pentobarbital and isoflurane on the responses of cutaneous mechanoreceptive afferents.

In experiments on Rhesus macaques (*Macaca mulatta*), a small dose of pentobarbital results in a considerable depression of both evoked potentials [2] and stimulus-evoked firing rates of cortical neurons [3]. Similarly, the addition of isoflurane to pre-existing anesthesia (nitrous oxide/narcotics) causes marked attenuation of motor evoked potential amplitudes [4], and somatosensory evoked potentials in response to electrical stimulation of the median nerve decrease in a dose-dependent manner with increasing concentrations of isoflurane [5,6]. When directly compared, the evidence suggests that isoflurane has a more profound impact on cortical responses than does pentobarbital [7].

Although the evidence that these two anesthetics affect cortical responses is unequivocal, their documented effects on the peripheral nervous system are not. Indeed, anesthetics appear to produce combinations of excitatory and depressant effects that are dependent on the preparation and/or type of neurons investigated. For example, pentobarbital reduces the amplitude of the early and late components of compound action potentials, associated with A and C fibers, respectively [8]. However, responses were evoked through electrical stimulation of the nerve, so could not be attributed to mechanoreceptive afferents. Isoflurane activates C fiber nociceptors, depresses A-δ mechanoreceptive fibers [9] and results in a decrease in receptive field size and/or suppression of responses of spinal cord neurons [10] without affecting conduction velocities [10,11]. Isoflurane also reduces neuronal excitability in a small subset of trigeminal ganglion neurons of decerebrate guinea pigs [12] and of wide-dynamic-range and nociceptive specific neurons in the lumbar dorsal horn of goats [13].

In the present study, then, we sought to further characterize the effects of anesthesia on the responses of mechanoreceptive afferents. We used sinusoidal vibrations to compare the effects of two anesthetic regimes – isoflurane and pentobarbital – on afferent excitability because the spiking behavior of afferents elicited by these stimuli has been extensively characterized [14]. Indeed, as afferents produce highly periodic responses to sinusoidal vibrations, using these stimuli allowed us to investigate the effects of anesthesia not just on the strength but also on the temporal patterning in afferent responses. First, we wished to determine whether changes in cortical responses caused by anesthesia might be due to its effects on afferent responses. Second, we wished to determine whether isoflurane was a suitable alternative to pentobarbital in neurophysiological experiments.

## Methods

### Neurophysiological procedure

All experimental procedures complied with the NIH Guide for the Care and Use of Laboratory Animals and were approved by the Animal Care and Use Committee of the University of Chicago. Experiments were conducted on 21 anesthetized Sprague–Dawley rats (*Rattus norvegicus*), weighing between 330 and 632 g. Anesthesia was induced with 4% inhalant isoflurane in an induction chamber; the animal was removed after loss of its righting reflex. To maintain anesthesia, 1.5-2% isoflurane in 100% oxygen was delivered via nose cone mask. Adapting a technique developed for primates [15], an incision roughly 4 cm long was made just posterior to the right femur, running parallel to the femur. The edges of the incision were then sutured to a plastic ring that was firmly secured to the table in order to create a skin-flap pool. The sciatic nerve was exposed by separating the biceps femoris muscle from the gluteus superficialis muscle in its aponeurosis. After isolating the sciatic nerve from the adjacent muscles, an 18-mm-diameter laryngeal mirror was positioned under the nerve, and the basin formed by the skin flap was filled with saline to prevent the nerve from drying. The epineurium and perineurium of the nerve were peeled back sequentially with microforceps. A small nerve bundle was cut from the proximal portion of the nerve and placed on the mirrored platform. The saline in the skin-flap pool was then replaced with mineral oil to avoid signal shunting, and the nerve bundle (~250 μm in diameter) was carefully teased into smaller fiber bundles. Each of these smaller bundles was wrapped individually over a hooked silver wire recording electrode. Bundles were subdivided until action potentials from a single fiber were clearly isolated. Single unit potentials were amplified with an AC/DC differential amplifier (Model 3000, A-M Systems Inc., Sequim, WA), filtered using an equalizer (Ultragraph PRO FBQ3102, Behringer, Willich, Germany) and displayed using custom-made spike sorting software. Responses were elicited by gently brushing the rat paw with a cotton-tipped applicator. Once a single unit was isolated, the stimulating probe (diameter = 1 mm) was fixed using cyanoacrylate to the point of maximum sensitivity (or hot spot) of its receptive field on the paw.

The stimulating probe was driven by a vibration exciter (Type ET-132-2, Labworks Inc., Costa Mesa, CA) driven by a linear power amplifier (Type PA-138, Labworks Inc., Costa Mesa, CA). The input voltage to the amplifier, under computer control, was delivered digitally by a multifunction data acquisition card (PCI-6229, National Instruments, Austin, TX; maximum single-channel output rate = 833 kHz; output rate used = 100 kHz). Probe movements were monitored using an accelerometer (Type 8702B50M1, Kistler Instrument Corporation, Amherst, NY) with a dynamic range of ±50 g. We used the side connector version of the accelerometer to facilitate mounting the accelerometer between the electrodynamic transducer and the stylus that delivered the stimulus to the skin. The threaded stud on the top of the accelerometer was mounted directly to the armature of the vibration exciter, and the stud of the stylus was attached to the bottom of the accelerometer. The output of the accelerometer was amplified and conditioned using a piezotron coupler (Type 5134A, Kistler Instrument Corporation, Amherst, NY), then read into the computer using a multifunction data acquisition card (PCI-6229, National Instruments, Austin, TX; maximum input rate = 250 kHz; input rate used = 100 kHz).

Units were classified as rapidly adapting (RA) or slowly adapting (SA) based on whether they produced transient or sustained responses to a sustained indentation. RA fibers were further subclassified as type I, having small and well-defined receptive fields, or type II, having large and poorly defined receptive fields. RA-II afferents, also known as Pacinian (PC) fibers, are also exquisitely sensitive to vibrations at higher frequencies and produce a vigorous response to air puffs.

### Experimental design

Each experimental block, lasting 5 minutes, consisted of 10 repeated presentations of sinusoidal stimuli, each lasting 2 seconds, that varied in frequency and amplitude. The interstimulus interval was 0.1 second. Three different frequencies (25, 50, and 100 Hz) were tested, with 5 intensity levels at each frequency, spaced logarithmically (100, 177.8, 316.2, 562.3, and 1000 μm, peak-to-peak, for 25 Hz; 50, 88.9, 158.1, 281.2, and 500 μm for 50 Hz; 25, 44.5, 79.1, 140.6, and 250 μm for 100 Hz). The sinusoidal stimuli were ramped up and down with ramps lasting 50 ms.

We recorded the responses of a single unit across two experimental blocks, separated by 30 minutes. In the control condition, both experimental blocks were recorded while the rat was under isoflurane anesthesia. This condition was included to assess the non-specific effects of prolonged stimulation on afferent responses. We could then quantify the degree to which responses during the second block were different from those in the first block and compare this difference to its counterpart in the experimental condition.

In the experimental condition, the first experimental block was identical to that in the control condition (i.e., the rat was under isoflurane anesthesia). After the first block, pentobarbital was injected intraperitoneally (45 mg/kg) and, 20 minutes later, isoflurane was discontinued. Oxygen administration was continued throughout the second block. After the end of the second experimental block, the isoflurane was restarted to prevent the animal from waking (pentobarbital anesthesia lasts approximately 20–60 minutes in rats, see ref. [16]), and the animal was euthanized by intraperitoneal injection of pentobarbital sodium and phenytoin sodium (Euthasol, Virbac Corporation, Fort Worth, TX) followed by exsanguination.

We adopted this experimental design (isoflurane anesthesia followed by either isoflurane or pentobarbital anesthesia) because recovery from pentobarbital anesthesia is very slow and somewhat unpredictable. It was therefore impractical to counterbalance the order in which anesthesia was delivered. The statistical analysis, described below, allowed us to disentangle the effects of the two anesthetics on the strength and patterning in neuronal responses.

### Statistical analyses

Analyses were carried out using MATLAB (Mathworks Inc., Natick, MA). The effects of the different anesthesia regimes on afferent firing rates were analyzed using a three-way analysis of variance (ANOVA) with group (control or experimental), block number (1 or 2), and vibratory stimulus (one of three frequencies combined with one of five intensity levels) as factors. The critical factor, however, was the interaction between group and block number: Indeed, this interaction determines whether the difference (if any) between the responses in the first and second block is greater in the experimental or in the control group, and thus gauges the statistical reliability of the effect of anesthesia on afferent responses.

We also wished to assess whether anesthesia affected the temporal structure of afferent responses. Indeed, mechanoreceptive afferents exhibit highly stereotyped responses to sinusoidal stimuli; specifically, they produce one or a burst of action potentials within a restricted phase of each stimulus cycle, a patterning dubbed “entrainment.” We sought to compare the effects of the two anesthetic regimes on afferent entrainment to sinusoidal stimulation using vector strength [17] as a measure of entrainment. Each spike can be considered as defining a vector of unit length with a phase angle *θi* (0≦*θi* < 2π) by taking *θi* = 2π *f t*_*i*_ (mod 2π), where *t*_*i*_ is the timing of the spike, and *f* is the stimulation frequency. Vector strength *r* is given by:

$$r=\frac{1}{n}\sqrt{{\left(\sum_{i=1}^{n}\cos\mathrm{\theta i}\right)}^{2}+{\left(\sum_{i=1}^{n}\sin\mathrm{\theta i}\right)}^{2}}$$

Vector strength *r* takes on the value of 1 if all of the spikes occur precisely at the same phase in each stimulus cycle and a value of 0 if spikes occur uniformly over the stimulus cycle. We tested whether anesthesia type had a significant effect on the vector strength using an ANOVA with group, block number, and vibratory stimulus as factors. Again, the critical factor was the interaction between group and block number.

## Results

**Figure 1:**
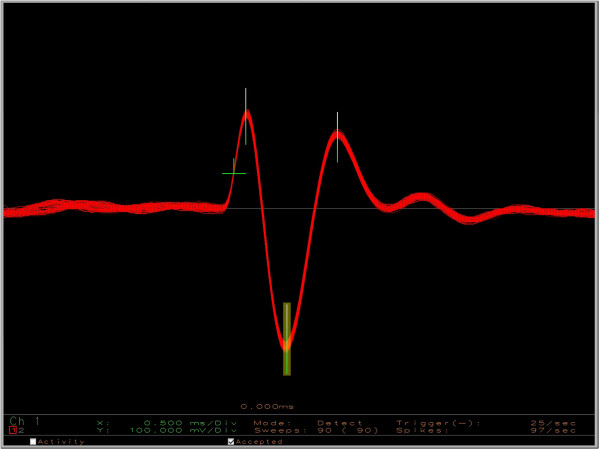
**Neurophysiological recordings.** An illustration of 90 (2-ms-long) action potential traces from a single unit displayed using custom-made spike sorting software (range of full screen: ±1 V). The signal to noise ratio is very high for these recordings.

**Figure 2:**
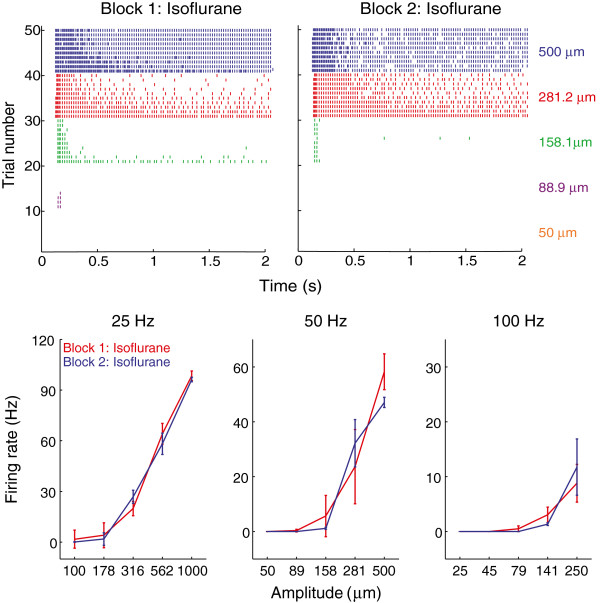
**Typical responses of a single unit under anesthesia with isoflurane only.** Top: Raster plots of the responses to 50-Hz stimulation in first (left) and second (right) blocks. Each row on the raster plots represents the spike train of the single unit corresponding to a given stimulus lasting for 2 seconds. Stimuli were presented ten times at each of five amplitudes (each indicated by a different color; at this frequency, the faintest stimulus did not elicit a response in this afferent). Bottom: Mean firing rates as a function of amplitude at the three frequencies tested. Each point indicates the average firing rate over 10 repetitions of each stimulus. Error bars denote the standard deviation. Note that we observed entrainment plateaus in a subset of fibers [14], but these were relatively uncommon given how sparsely amplitudes were sampled at each frequency.

**Figure 3:**
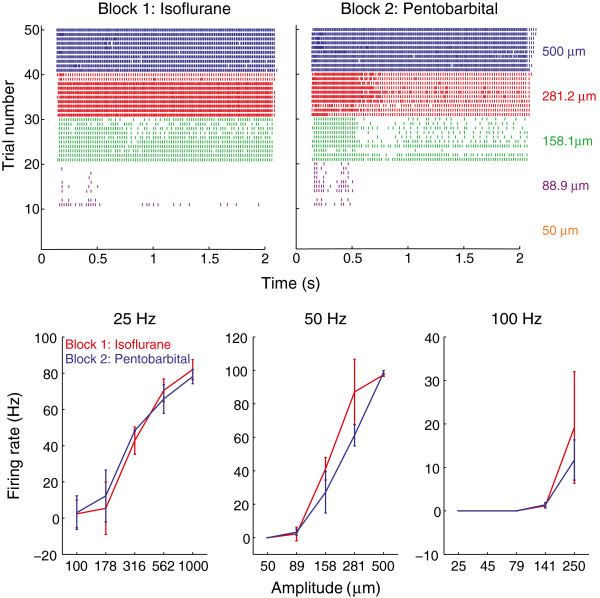
**Typical responses of a single unit under anesthesia with first isoflurane and then pentobarbital.** Top: Raster plots of the responses to 50-Hz stimulation under isoflurane (left) and pentobarbital (right). Each row on the raster plots represents the spike train of the single unit corresponding to a given stimulus lasting for 2 seconds. Stimuli were presented ten times at each of five amplitudes. Bottom: Mean firing rates as a function of amplitude at the three frequencies tested. Error bars denote the standard deviation.

We recorded the responses of 21 single units (11 control group, 10 experimental group; 18 type I RA fibers, 3 SA fibers, see Figure 1 for sample spike traces). Given the small number of SA fibers, we pooled data across fiber types, verifying that the two fiber types did not exhibit qualitatively different reactions to anesthesia. Figure 2 shows the mean firing rate of a single unit under anesthesia with isoflurane in the first and second experimental block (control condition). For this neuron, the responses were nearly identical in the two blocks. Figure 3 shows the mean firing rate of a (different) single unit under anesthesia with the first experimental block carried out under isoflurane anesthesia and the second under pentobarbital anesthesia (experimental condition). Again, the responses in the two blocks were not significantly different.

Because responses to vibratory stimuli vary widely across afferents, we normalized the firing rates of each afferent to then compare the effects of anesthesia across the entire population. Specifically, individual responses were transformed by subtracting from each response (i.e., the firing rate on each trial) the mean and dividing by the standard deviation of the responses across all stimulus conditions and experimental blocks (so that differences in firing rate across blocks would be preserved). This normalization removes any differences in the overall mean and standard deviation of the firing rates across afferents, leaving only differences across experimental blocks.

**Figure 4:**
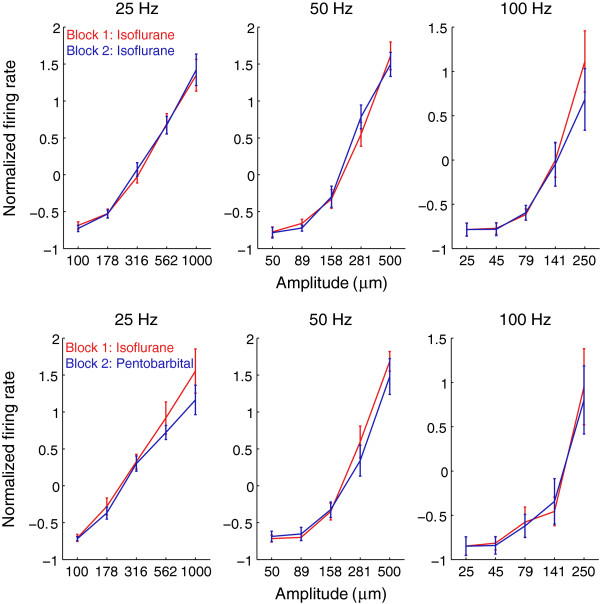
**Effect of anesthesia on response strength over the population.** Top: Normalized firing rates for the control group. Each point indicates the average normalized firing rate across single units of the same block under the same condition of stimulus. Error bars denote the standard error of the mean. Bottom: Normalized firing rates for the experimental group.

Figure 4 shows the normalized firing rates, averaged across all afferents, in the first block and second block, in the control and experimental conditions, respectively. Again, there was no systematic difference in firing rate between blocks for either group.

**Figure 5:**
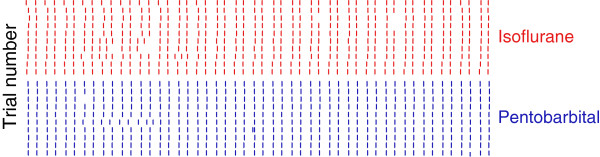
**Entrainment of a single unit recorded in the experimental condition.** Raster plots of the response evoked by 50-Hz, 500-μm sinusoidal vibration delivered for 0.5s.

**Figure 6:**
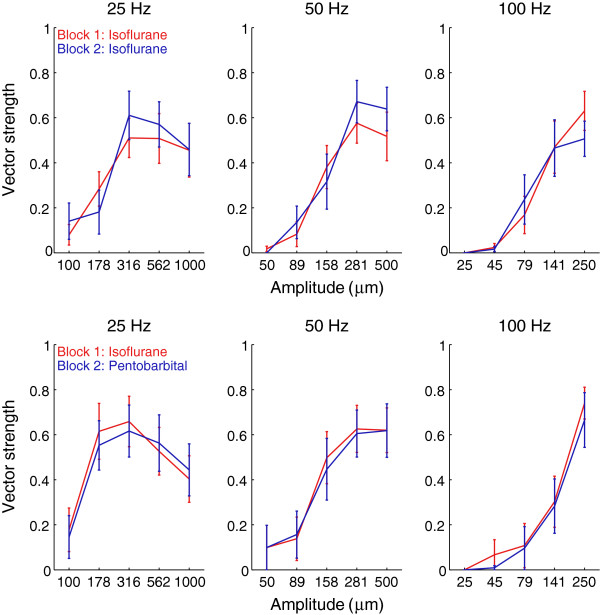
**Effect of anesthesia on the temporal structure of the response over the population.** Top: Entrainment in the control condition. Each point indicates the mean vector strength across single units of the same block under the same condition of stimulus. Error bars denote the standard error of the mean. While the degree of entrainment is highly variable across afferents (as indicated by the large error bars), it is consistent across blocks. Bottom: Entrainment in the experimental group.

We also investigated the effects of anesthesia on the temporal structure of the response. Figure 5 shows the responses of a single afferent to a 50-Hz sinusoid under isoflurane anesthesia (red) and pentobarbital anesthesia (blue). As can be seen, the fine structure of the response is virtually identical under the two anesthetic regimes. Figure 6 shows the strength of entrainment of afferents in the first block and second block in the control and experimental conditions, respectively. There was no systematic effect of anesthesia on the temporal structure of the response across the afferent population (see ANOVA results below).

As expected, there was a significant effect of vibratory stimulus on both firing rate (*F*(14, 584) = 103.2, p < 0.001) and vector strength (*F*(14, 584) = 25.7, *p* < 0.001). There was no significant difference in firing rate (*F*(1,584) = 1.33, *p* > 0.2) or entrainment (*F*(1,584) = 0, *p* > 0.5) across blocks. Importantly, however, the interaction between block number and group (control or experimental) was non-significant for both firing rate (F(1,584) = 0.5, p > 0.4) and vector strength (F(1,584) = 0.5, p > 0.05). In other words, the difference in response between the first and second blocks was not different when pentobarbital was used in the second block than when isoflurane was used. That is, anesthesia type did not have any effect on either the strength of or temporal patterning in the response. In conclusion, while the two anesthetic regimes may have affected the neuronal response, their effects were statistically indistinguishable.

## Discussion

The main result in the present study is that isoflurane and pentobarbital do not differentially affect the responses of mechanoreceptive afferents. Indeed, both the strength of the afferent response and its fine temporal structure were statistically indistinguishable in the two conditions. Thus, differences in the effects of the two anesthetics on cortical responses cannot be attributed to differences in input from the periphery. Furthermore, the majority of neurophysiological experiments investigating the responses of mechanoreceptive afferents have been carried out using pentobarbital. The results of the present study suggest that isoflurane, a much safer anesthetic [18], yields identical results as does pentobarbital and so should be preferentially used in such experiments. Note that, although these experiments were carried out on cutaneous afferents of rats, mechanoreception in rodents, felines, and primates is highly homologous [19], so our results are likely to generalize across these mammalian orders.

### Residual isoflurane

We wished to compare the effects of the two anesthetics on the responses of individual afferents, so it was necessary to maintain the isolation of the afferent signal through both experimental blocks and the inter-block interval. There was therefore a trade-off between extending the duration of the inter-block interval (to allow isoflurane to be eliminated) and maintaining the quality of recording from the neuron. We chose that duration to be 30 minutes. According to published data [20], approximately 52-72% of arterial isoflurane has been eliminated at this point in time in rats after an average of 4 hours of exposure to 1.8% isoflurane. In the current study, animals had been under anesthesia for an average of 2.5 hours by the end of the first experimental block, so the above estimate of residual isoflurane is likely an overestimate. In the experimental condition, responses in the second block thus reflected both the effects of pentobarbital and of some residual isoflurane. One possibility is that isoflurane has an effect on afferent responses that is relatively dose-independent. According to this view, measurements in the first and second block reflect an equivalent effect of isoflurane, and pentobarbital does not exert any additional effect on afferent responses.

### Molecular mechanisms

At the periphery, isoflurane has been shown to inhibit neuronal Ca^2+^ channels through enhancement of current inactivation in rat dorsal root ganglion (DRG) neurons [21] but the behavioral relevance of these peripheral effects is unclear [22]. Pentobarbital may also lead to a reduction in the excitability of DRG neurons by suppressing the purinergic P2X receptors [23] and by activating Cl^-^ channels [24]. No study to date has investigated the extent to which these molecular mechanisms are pertinent to mechanoreceptive afferent neurons as the DRG comprises a wide variety of neurons, ranging from mechanoreceptive to thermoreceptive and nociceptive. Our results suggest that, to the extent that pentobarbital and isoflurane have an effect on mechanoreceptive afferent responses, their effects seem to be identical. Given the similarity in the responses measured in (non-anesthetized) humans using microneurography and those measured in macaques anesthetized with pentobarbital [25,26], it is likely that isoflurane and pentobarbital have negligible effects on the responses of mechanoreceptive afferents.

## Conclusions

We conclude that isoflurane and pentobarbital have identical (and probably negligible) effects on the responses of mechanoreceptive afferents. Indeed, both the strength and temporal structure of afferent responses to vibratory stimuli are indistinguishable under the two anesthetic regimes.

## Competing interests

The authors declare that they have no competing interests.

## Authors’ contributions

JWC, AIW and SJB designed the experiments, JWC collected the data, JWC and AIW analyzed the data, JWC, AIW and SJB drafted the manuscript. All authors read and approved the final manuscript.

## Pre-publication history

The pre-publication history for this paper can be accessed here:

http://www.biomedcentral.com/1471-2253/13/10/prepub
